# A study to assess the pharmacological agent potential of gold nanoparticles and their effects on human cancer cells and hospital pathogens using *in vitro* methods

**DOI:** 10.3389/fphar.2024.1498734

**Published:** 2025-01-06

**Authors:** Ayşe Baran

**Affiliations:** Department of Biology, Mardin Artuklu University Graduate Education Institute, Mardin, Türkiye

**Keywords:** anticancer, antimicrobial, Au NPs, broccoli, pharmacological agent

## Abstract

Interest in metal nanoparticles synthesised using green methods is growing steadily. Metal nanoparticles can be synthesised inexpensively and effortlessly using extracts derived from different plants and their diverse components. Gold nanoparticles (Au NPs) were rapidly synthesised from Broccoli (*Brassica oleracea* L.) agricultural waste using a novel, cost-effective, and eco-friendly method in this work. Analysed data from various techniques including UV-vi, TEM, FESEM, XRD, AFM, FTIR, TGA-DT, EDX, and DLS to assess the properties of the synthesised Au NPs. The characterisation data revealed that the Au NPs had a peak absorbance at 553.10 nm, a surface charge of −19.7 mV, an average hydrodynamic size of 78.75 nm, a monodisperse spherical shape, and were found to be stable. The inhibitory effects of Au NPs with these properties on hospital pathogens and human cancer cells were evaluated by microdilution, disk diffusion and MTT techniquesAs a result of the findings, it was determined that Au NPs have antibacterial, antifungal and anticancer potential as pharmacological agents under *in vitro* conditions.

## 1 Introduction

Nanoparticles are highly desirable materials due to their distinctive features, including attraction, conductivity, surface charge, enormous surface area, and adjustable energy bands. Nanoparticles (NPs) typically range in size from 1 to 100 nm. Acquiring these items is continuously advancing as a prominent field in contemporary material sciences. Metal nanoparticles in these items are utilised in several fields including biomedical applications, sensors, electronics, optics, food, and cosmetics. Au NPs, a type of metal nanoparticles, are highly prized in medical research for their drug delivery, anticancer, and antibacterial capabilities. This is attributed to their exceptional optical characteristics, biocompatibility, and ease of functionalization (Parida et al., 2011; Velmurugan et al., 2014; Rautray and Rajananthini, 2020; Al-Mafarjy et al., 2024; Ejaz et al., 2024). There are numerous techniques such as biological, physical, chemical available for producing Au NPs. Green approaches are more favourable than physico-chemical methods. Toxic chemicals are not utilised in the production of green-synthesized Au NPs. Furthermore, green synthesis is a cost-effective technique with minimal energy consumption. The roads are simple and cost-effective, and they are environmentally beneficial. Au NPs produced using these methods have numerous benefits, including biocompatibility, high yield, and easy access to the synthesis source (Mythili et al., 2018; Patil et al., 2018; Korani et al., 2021). Green synthesis research can utilise different plant parts such leaves, flowers, roots, fruits, or the entire plant as a source for synthesis. Phytochemicals such phenolic acids and flavonoids in plant extracts work as active functional groups that reduce ionised +3-valent Au, leading to the creation of Au NPs and influencing their stability (Shankar et al., 2016; Manikandan et al., 2024; Oliveira et al., 2024; Rosyidah et al., 2024).

*Brassica oleracea* L. (Broccoli), a perennial herbaceous plant in the Brassicaceae family, is commonly eaten for its beneficial components. Broccoli is a beneficial plant rich in nitrogen-sulfur derivatives like glucosinolates and isothiocyanates, as well as polyphenols such as chlorogenic and sinapic acid derivatives, flavonoids, and health-promoting phytochemicals including potassium, selenium, and manganese. This plant grows in the Iran-Turan phytogeographic region. The plant thrives in areas with gravelly and rocky slopes, specifically in Bingöl, Ağrı, and Elazığ in Eastern Anatolia, Turkey. Broccoli shoots have the potential to reach a maximum height of 40 cm. Although the lower portions of these plants are covered in leaves, the upper portions are devoid of foliage (Singh et al., 2018; Le et al., 2020).

Within this research investigation, it was aimed to synthesize Au NPs in an environmentally friendly way with the extract obtained from the agricultural waste parts of broccoli with a new approach in the agricultural cultivation area in Diyarbakır Bismil region, then to determine their properties and evaluate their usability as medical agents.

## 2 Materials and methods

### 2.1 Preparation of extract and chlorauric acid solution for synthesis medium

In November 2023, agricultural waste from broccoli (After harvesting broccoli, remove all above-ground parts except the crown and stem) were sent to the agricultural production region in Diyarbakır Bismil Region. Initially, use tap water to wipe off mud and other debris. The remaining particles were removed. Subsequently, multiple cleaning procedures were carried out using distilled water. The cleansed agricultural waste were chopped into small fragments with a knife and subsequently crushed in a mortar. 50 g of this substance were weighed. And left to sit for 20 min at 50°C with 250 mL of distilled water, without any drying process. The extract was promptly cooled to room temperature and then filtered without delay after the drying process. The extract was acquired for the purpose of synthesising Au NPs.

An Alfa Aesar branded solid compound of Tetrachloroauric (III) acid trihydrate (HAuCl_4_) from Kandel, Germany was utilised as the metal source in the production of Au NPs. A metal solution with a concentration of 2 mM (mM) has been formed from this chemical for the production of Au NPs.

### 2.2 Synthesis of Au NPs

**Scheme 1:**
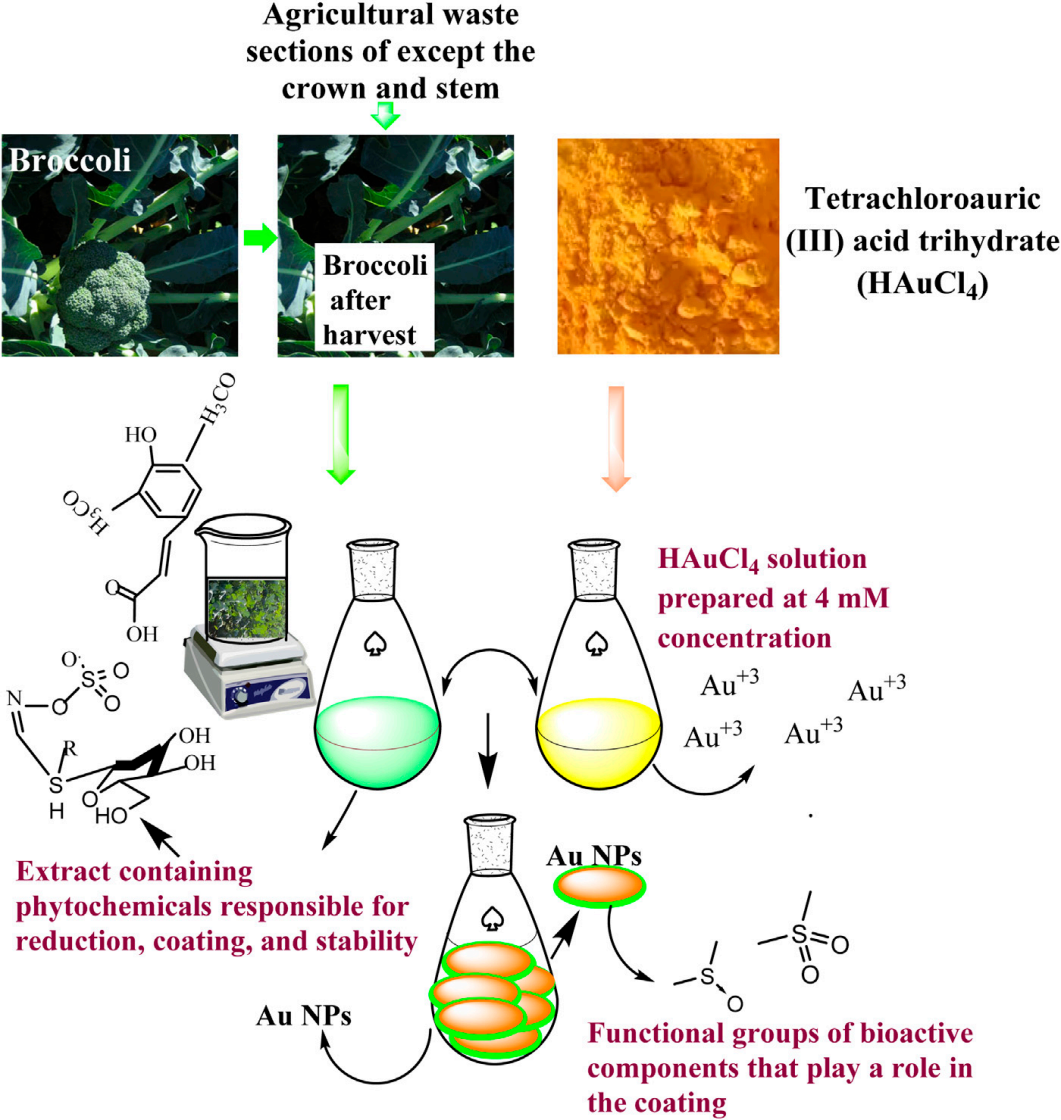
Representative scheme of the synthesis mechanism of Au NPs by extract components obtained from agricultural parts of Broccoli.

The extract from agricultural waste sections of broccoli was combined with a 2 mM HAuCl4 solution in a 1:1 ratio. The mixture was stirred at 100 revolutions per minute (rpm) at a temperature of 45°C for 20 min. The colour change that occurred within 5 minutes and the following colour alterations were noted (Scheme 1). Samples were collected from the synthesis medium at specific phases, and the absorbance at their maximum wavelength was determined. Au NPs production and presence were characterised using this. To further characterise the colloidal Au NPs generated from the synthesis and their use as medicinal agents, the precipitation procedure was carried out at 6,000 rpm for 10 min. The precipitate obtained was rinsed with distilled water multiple times and subsequently dehydrated in an oven at 70°C.

### 2.3 Characterization of Au NPs

A Perkin ElmerOne UV-vis spectrophotometer was utilised to show the surface plasma resonance (SPR)-induced creation of Au NPs produced by the extract and their specific peak absorbance wavelengths. The study analysed the generation and existence of Au NPs by measuring their maximum wavelength absorbances within the 300–800 nm range.

Measurements from a Shimadzu TGA-50 TGA-DTA and a Marven DLS were utilised to assess the mass losses, surface charges, and hydrodynamic size distributions of Au NPs produced from broccoli agricultural waste components in response to increasing temperature changes.

A Park System XE-100AFM, JeolJem010 TEM, and FEI Quanta 250 FEG FE-SEM instruments were utilised to examine the morphological characteristics and structures of Au NPs. The micrographs obtained by the devices were used to analyse the morphological appearance of Au NPs. The crystal structures and elemental compositions of the particles produced during synthesis by the extract components were analysed using data from measurements taken by a Rigaku Miniflex 600 model XRD and a RadB-DMAXIIEDX. The crystal nanosizes were determined using the Full Width at Half Maximum (FWHM) values of the peak acquired from the X-ray Diffraction (XRD) data, applying the Debye-Scherrer equation (Li et al., 2024).

The functional groups of the phytochemicals responsible for forming, coating, and stabilising AuNPs by reducing Au+3 in the synthesis medium from broccoli agricultural waste were identified using spectra obtained with a Perkin Elmer One FTIR. Spectra in the region of 4,000–500 cm^−1^ from the extract and liquid portions produced during the synthesis were utilised for this analysis.

### 2.4 Applications of Au NPs in medicine

#### 2.4.1 Antimicrobial activities

The study investigated the antibacterial properties of Au NPs produced from broccoli agricultural waste on pathogenic strains by the Micro Dilution method (Finegold et al., 1967; Zapata and Ramirez-Arcos, 2015). The application found the Minimum Inhibitory Concentrations (MIC) at which reproduction was suppressed. The microorganisms utilised in experimental investigations are listed in Table 1. The pathogenic organisms were obtained from the Microbiology Laboratory at Dicle University Scientific and Technological Research Centre in Diyarbakır, Turkey.

**TABLE 1:** Strains of microorganisms and the qualities of those strains that are used in the application of antimicrobial effects.

Microorganisms		Structures of the cell wall	Number of the ATTC
Bacteria	*Escherichia coli* (*E. coli*)	—	11229
	*Pseudomonas aeruginosa* (*P. aeruginosa)*	—	27833
	*Bacillus subtilis* (*B. subtilis*)	+	11774
	*Staphylococcus aureus* (*S. aureus*)	+	25923
Fungi (Yeast)	*Candida albicans* (*C. albicans*)	+	10231

In experimental studies, each strain was propagated under optimum conditions. The bacterial strains were cultured on nutrient agar and *C*. *albicans* on sabora dextrose agar medium, then incubated at 37°C overnight for growth. Microorganism colonies were checked. Solutions complying with the McFarland standard 0.5 turbidity criterion were prepared by using microorganism colonies grown on the medium plates (Zapata and Ramirez-Arcos, 2015). Antimicrobial efficacy was evaluated using microdilution assays conducted in 96-well microplates. Muller Hinton liquid medium was added to the wells for each bacterial strain. Roswell Park Memorial Institute (RPMI) 1640 broth was put in to the wells for the *C. albicans* strain. Two wells in the microplate have been allocated for growth control and sterilisation control. Au NPs with different concentrations were introduced to the wells, and then microdilution was performed. Microorganisms produced using the McFarland 0.5 standard (1.5 × 10^8 CFU/mL) were transferred to microplate wells. The same microdilution procedure was used with a 2 mM HAuCl_4_ solution as the metal source in the synthesis medium and antibiotics, for comparative analysis. Fluconazole antibiotics were employed for *C. albicans* strains, vancomycin for gram-positive bacteria strains, and colistin antibiotics for gram-negative bacteria strains. The microdiluted microplates were incubated at 37°C for 24 h to assess the interactions of all strains with Au NPs, HAuCl_4_ solution, and antibiotics utilised in the experiment. After the interval ended, the microplate wells were examined for growth. The concentration at the well where growth initiation occurred was identified as the Minimum Inhibitory Concentration (MIC) that ended growth. Experiments were conducted in triplicate (n = 3).

The antimicrobial activity of the synthesised Au NPs on Gram (−) *E. coli* ve Gram (+) *S. aureus* (Table 1) bakterileri ile birlikte *C. albicans* ATTC 10231 were also examined using the Disc Diffusion method (Hudzicki, 2012). Microorganisms were cultivated in suitable settings, under optimal conditions, in an incubator at 37°C. Mueller-Hinton agar from Merck was utilised as the medium to examine the antibacterial action. Suspensions of each microbe strain were formulated to conform to the McFarland 0.5 turbidity standard (1.5 × 10^8 CFU/mL) (Hudzicki, 2012; Zapata and Ramirez-Arcos, 2015). Following the impregnation of sterile discs with Au NPs and a control solution of HAuCl_4_, each microorganism was grown on Mueller-Hinton agar medium. Prepared discs were positioned in Petri dishes at preset intervals. To facilitate interaction between the substances applied to discsand the microorganisms, they were maintained at 37°C for 24 h. Upon the conclusion of the time, observations were conducted, and the inhibition zones were quantified in millimetres utilising a digital calliper (Mitutoyo). Experiments were conducted in triplicate (n = 3).

#### 2.4.2 Anticancer effects of Au NPs

The impact of Au NPs produced from broccoli agricultural waste on healthy cells and three types of cancer cells was assessed using the MTT [3-(4,5-dimethylthiazol-2-yl)-2,5-diphenyltetrazolium bromide] assay (Hatipoglu et al., 2023). Experiments were conducted at Dicle University Scientific Research Centre, Cell Culture Laboratory, located in Diyarbakır, Turkey. Table 2 shows that Colorectal Adenocarcinoma (Caco-2), Glioblastoma (U118), and Human Ovarian Sarcoma (Skov-3) cancer cell lines were utilised. The cytotoxic effects of Au NPs on the healthy cell line Dermal Fibroblast (HDF) were evaluated in practice.

**TABLE 2:** Healthy and cancer cell lines utilised for assessing cytotoxic effects using the MTT technique.

Cell lines	Histopathology	Cancer-healthy
Skov-3	Cancer	Ovarian Cancer Epithelium
U118	Cancer	Brain Cancer Epithelium
Caco-2	Cancer	Kolorektal Adenokarsinom Epitel
HDF	Healthy	Skin Fibroblast

CaCo-2, HDF, and U118 cell lines were propagated in 75 t-flasks in DMEM (Dulbecco’s Modified Eagle) medium. Skov-3 cells used in the application were grown and used in RPMI medium in 75 tissue culture flasks. All cultured cells were incubated for proliferation at a temperature of 37°C oven where 95% air, humidity, and 5% CO_2_, conditions were optimally adjusted. At the end of the period, cells checked by Hemocytometer and reaching 80% confluency were used in experimental studies. These cells, suspended in different concentrations, were transferred to 96-well microplate wells for MTT application. The microplate wells were incubated for 24 h. Au NPs made at various concentrations were introduced into the microplate wells at the conclusion of the time, and the cells were incubated at 37 °C for 24–48 h to facilitate interaction. Following incubation, MTT solution was introduced to the microplate wells and allowed to react treated with dimethyl sulfoxide (DMSO) for a duration of 3 h and 15 min. The absorbance of the cell lines in the microplate wells was quantified at the end of the experiment using the Multi ScanGo Thermo equipment, which was configured to measure at a wavelength of 540 nm. Experiments were conducted in triplicate (n = 3). With the measured absorbances, the concentrations of Au NPs that suppressed viability on the cell lines were calculated using the % viability threshold given below.

% viability=U/C*100

In equality; U = absorbance of cells interacting with Au NPs, C = absorbance of control cells without Au NPs (Baran, 2023).

## 3 Results and discussion

### 3.1 UV vis data of Au NPs

**FIGURE 1:**
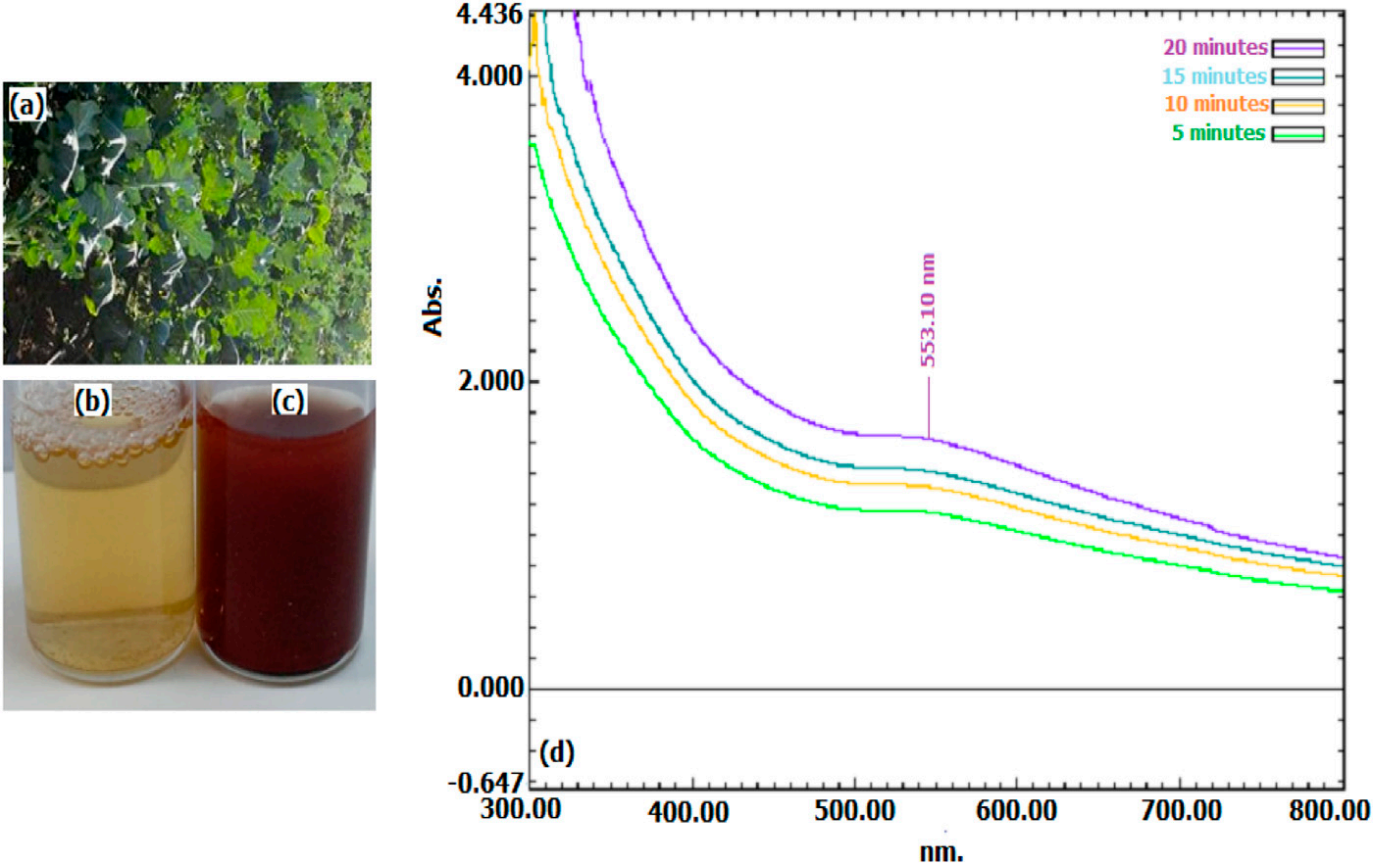
**(A)** Broccoli agricultural waste, **(B)** extractcolour change indicating the presence of Au NPs synthesised by **(C)**, and **(D)** maximum wavelength absorbances in UV-visible spectrum data.

30 min after combining the extract from broccoli agricultural waste parts with the HAuCl3 solution, the colour changed from pink to red. UV-vis maximum wavelength scans were conducted on samples taken from the reaction media to observe the colour change caused by the production of surface plasma resonance (SPR) of Au NPs, which lasts around 60 min. As seen in Figure 1, the production of Au NPs was characterized by measurements using absorbance readings recorded at 553.10 nm (Al-Radadi, 2023; Rashidipour et al., 2024; Rosyidah et al., 2024).

### 3.2 Morphologies and crystal structures of Au NPs

**FIGURE 2:**
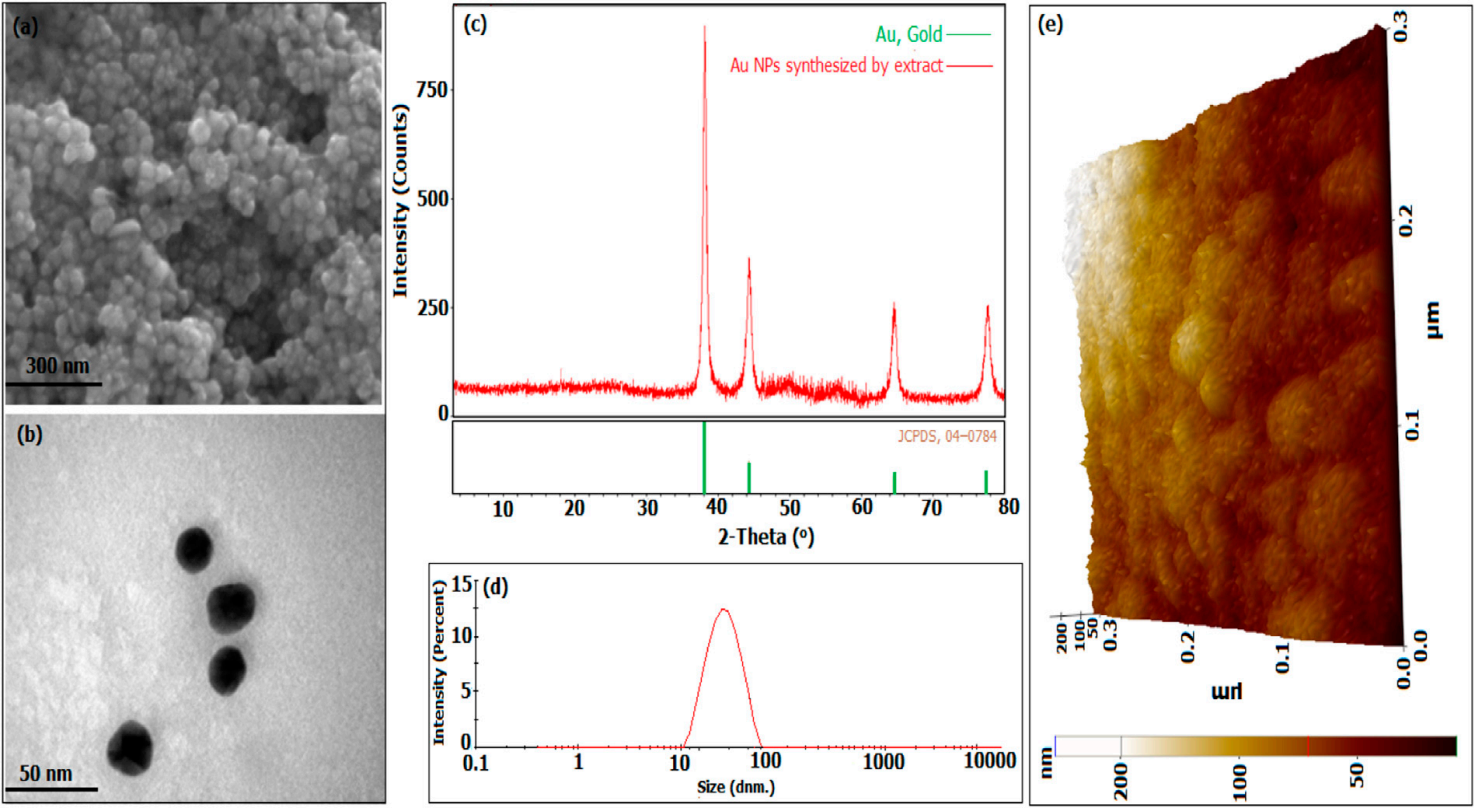
Morphological characteristics, crystal structures, and size distributions of Au NPs produced from broccoli agricultural waste extract: **(A)** Field Emission Scanning Electron Microscopy (FESEM) and **(B)** Transmission Electron Microscopy (TEM) images, **(C)** X-ray Diffraction (XRD) analysis, **(D)** Dynamic Light Scattering (DLS) data, and **(E)** Atomic Force Microscopy (AFM) image.

The morphological appearance and crystal structures of Au NPs synthesized through the extract were evaluated with measurement data obtained by FESEM (Figure 2A), TEM (Figure 2B), XRD (Figure 2C), DLS (Figure 2D), and AFM (Figure 2E). In Figures 2A, B, D, E, it was determined that they had a spherical appearance and exhibited a size distribution below 100 nm in the FESEM, TEM, and AFM micrograph findings, respectively (Adem et al., 2024). The crystal structures of the synthesised Au NPs were analysed in the data obtained within the 20–80 range of 2θ (Figure 2C). The Bragg’s angles expansions were used to determine it. The FVHM values of these degrees were analysed. The average crystal nanosize was calculated to be 62.42 nm using the Full Width at Half Maximum (FWHM) value of the highest peak (111) with the Debye-Scherrer equation. The data obtained from XRD analysis matched the synthesised Au NPs as per the Joint Committee on Powder Diffraction Standards (JCPDS) number 04–0784 (Akhil et al., 2024; Ejaz et al., 2024). Figure 2D showed that the synthesised Au NPs had an average size of 78.75 nm, falling within hydrodynamic size ranges below 100 nm. The synthesised material can be valuable for applications that necessitate Au NPs with a size distribution under 100 nm and a spherical shape. The hydrodynamic size distribution of Au NPs was determined to be 53.8 nm in a green synthesis investigation using DLS (Awad et al., 2019) In a synthesis study conducted with Satureja *Khuzistanica Jamzad* extract, it was stated that Au NPs had a spherical morphology (Rashidipour et al., 2024). A study using *Allium cepa* extract shown by TEM, SEM, and AFM pictures that Au NPs exhibit a spherical shape and have a size distribution under 100 nm (Ipek et al., 2023). The crystal pattern of Au NPs synthesised using Rheum ribes fruit peel extract supported the findings. The crystal nano dimensions were predicted to be 48.96 nm using Debye-shrerer equation (Baran, 2023).

### 3.3 Surface structures of Au NPs

**FIGURE 3:**
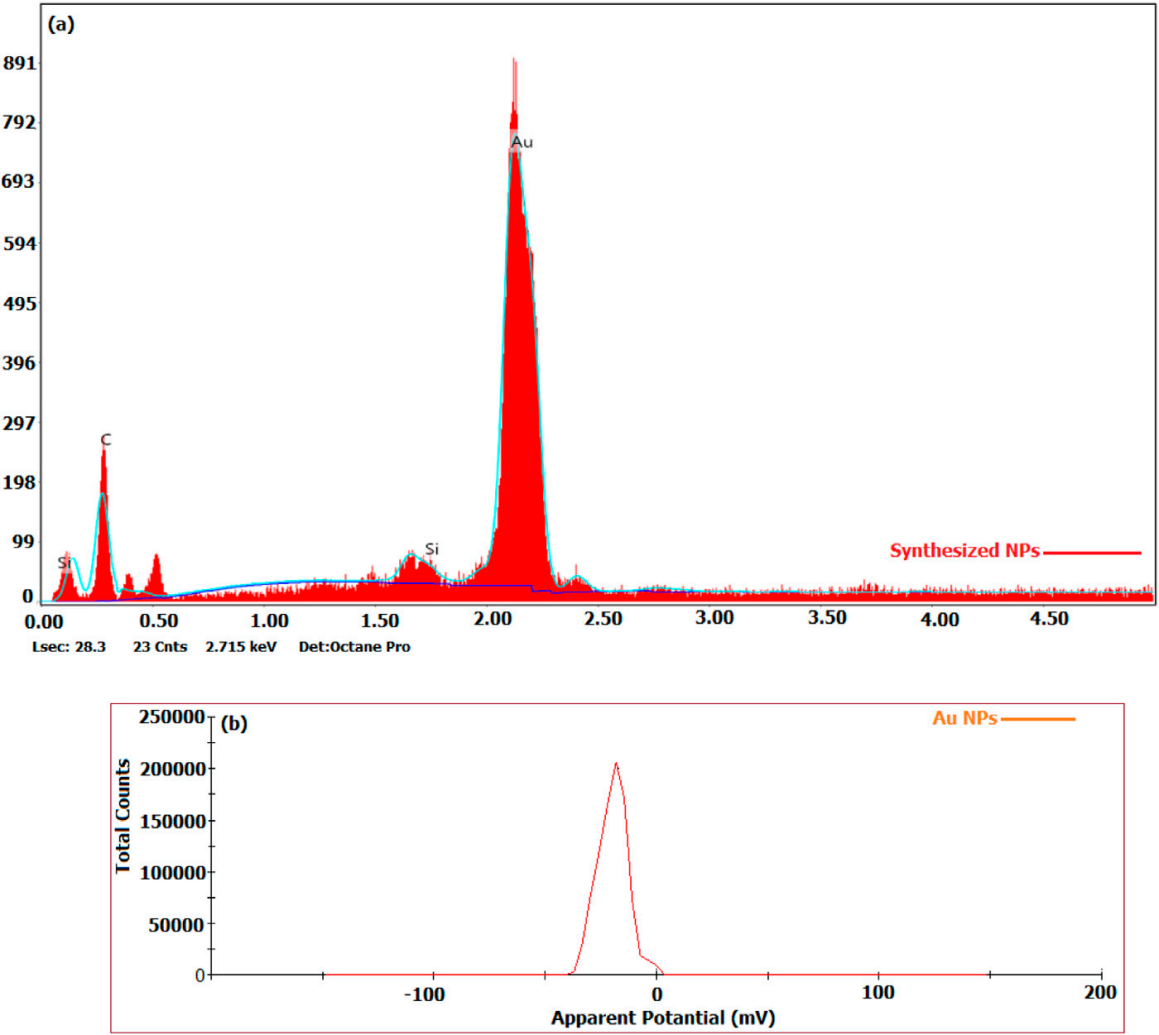
**(A)** Elemental compositions of particles produced by the extract, **(B)** Surface charge distributions obtained by DLS.

**FIGURE 4:**
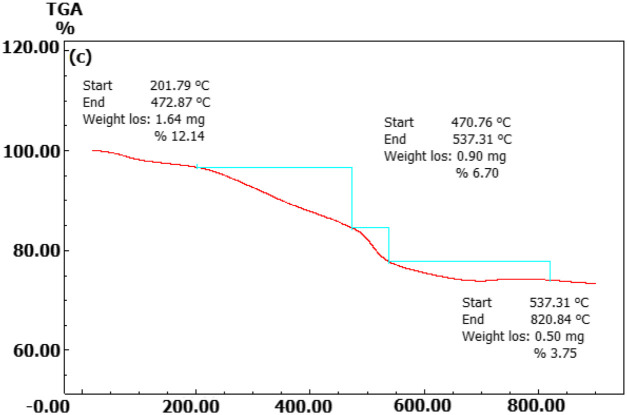
Temperature points at which synthesised Au NPs experience mass loss as determined by TGA-DT.

**FIGURE 5:**
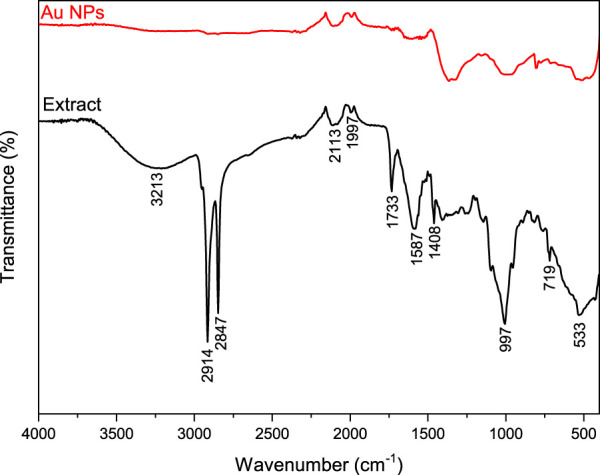
FTIR spectra of Au NPs and extract.

The elemental content of the particles synthesised from broccoli farm waste was analysed using the EDX data provided in Figure 3A. Figure 5’s element profile displayed prominent peaks indicating the presence of Au NPs. The EDX profile showed low peaks for O and C elements, which are associated with phytochemical components involved in coating Au NPs (Doan et al., 2020; Chen et al., 2021; Hosny et al., 2021; Mandhata et al., 2021; Rosyidah et al., 2024). The DLS zeta potential results presented in Figure 3B indicated that the average surface charge of the synthesised Au NPs was −19.7 mV. The research revealed that the surface charges of the synthesised Au NPs were exclusively negative. Understanding the negative surface charge is crucial for scenarios impacting stability, such as aggregation and fluctuation. Only the negative surface charge had a characteristic that favourably impacted stability in this discovery. If nanoparticles possess both negative and positive charges, they would lead to aggregation, resulting in adverse effects on different applications. Phytochemicals in the coating are the primary factor responsible for the negative surface charge. The surface charge distribution of Au NPs in a green synthesis investigation was found to be −19.3 mV (Giljohann et al., 2010; Khan et al., 2019; Webster et al., 2020). Figure 4 and Table 3 present the TGA-DT analysis results, which assessed the mass losses of the synthesised Au NPs as the temperature increased. It was noted in the data analysis that the mass losses of Au NPs took place within three distinct temperature intervals ranging from 25° to 1,000°C. The initial mass reduction, happening between 201.79 and 472.87°C, was caused by the elimination of adsorbed water. The mass losses observed in temperature ranges of 470.76–537.31°C and 597.31–820.84°C were caused by bioorganic molecules derived from phytochemicals. This discovery also revealed the existence of phytochemical substances produced by the coating around the nanoparticles (Doan et al., 2020; Sepahvand et al., 2020; Padalia and Chanda, 2021).

**TABLE 3:** Investigating the mass loss points and rates of Au NPs produced from broccoli agricultural waste at varying temperature increases.

Points of Mass loss	Loss of mass (%)	Temperature (^o^C)
First	12.14	201.79–472.87
Second	6.70	470.76–537.31
Third	3.75	597.31–820.84

### 3.4 FTIR data

FTIR data was utilised to assess the phytochemical components extracted from agricultural waste sections of broccoli in relation to functional groups. The active groups responsible for the synthesis and stability of Au^+3^ form of Au NPs, as well as their coating, were identified by analysing the frequency changes in the spectra of both the extract and the liquids obtained from the reaction shown in Figure 4. The alterations in the spectra observed at three places were utilised for assessment. The FTIR data presented in Figure 5 indicates that the shifts in the spectra at 3,213 cm^−1^, 2,914 cm^−1^, 2,847 cm^−1^, and 2,113 cm^−1^ suggest the involvement of phenol/alcohol groups (O–H) and alkene groups (–C=C–) in the bioreduction process. The spectral frequency shifts at 1,733, 1,587, and 1,408 cm^−1^ indicated that amine groups (^−^NO) were primarily responsible for the coating and stability (Usman et al., 2019; Babu et al., 2020; Donga et al., 2020).

### 3.5 Antimicrobial effects of Au NPs

**FIGURE 6:**
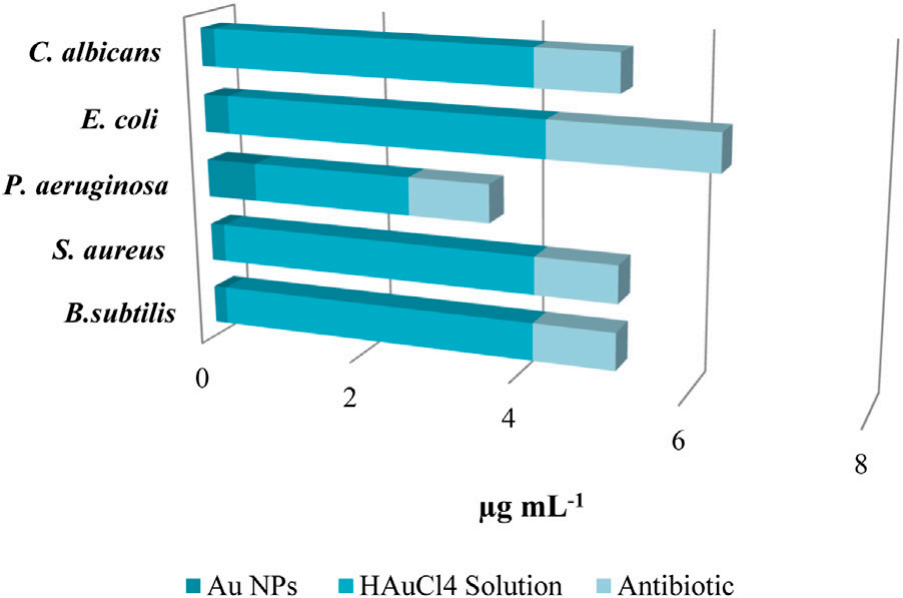
MIC values were determined by assessing the antibacterial activity of Au NPs on pathogenic strains, and were compared to the effects of HAuCl_4_ solution and antibiotics.

Microorganisms have evolved antibiotic resistance due to the inconsistent and unintentional administration of antibiotics, which remains a significant global issue. Efforts are underway to address this issue through various scientific endeavours. Studies have shown that metal nanoparticles can effectively address this issue (Nishanthi et al., 2019; Scarafile, 2016; Nieto-Argüello et al., 2022). This study utilised the microdilution approach to assess the minimum inhibitory concentrations (MICs) of synthesised Au NPs on pathogenic gram-negative and gram-positive bacteria as well as *C. albicans*. The effects of synthesised Au NPs were compared with the effects of suitable antibiotics for each strain and the HAuCl_4_ solution used for synthesis in the same application. Concentrations ranging from 0.16 to 0.64 μg mL^−1^ were shown to be efficient in inhibiting the growth of Gram-positive bacteria. The most significant impact at low concentrations was observed on *Bacillus subtilis*, *C. albicans*, and *S. aureus*, at 0.16 μg mL^−1^ and 0.18 μg mL^−1^, respectively. Significant inhibition was noted in the other strains. Furthermore, these concentrations of action were significantly lower compared to HAuCl_4_ solution and antibiotics (Table 4 ve Figure 6).

**TABLE 4:** MIC amounts at which the antibacterial effects of the synthesized Au NPs, antibiotics and HAuCl_4_ solution on the growth of microorganisms occur (n = 3, X̅ ± Sx̅).

Microorganisms	Au NPs µg mL^−1^	HAuCl_4_ solution µg mL^−1^	Antibiotic µg mL^−1^
*B. subtilis*	0.16 ± 0.02	4.00 ± 0.50	1.00 ± 0.16
*S. aureus*	0.18 ± 0.02	4.00 ± 0.63	1.00 ± 0.16
*P. aeruginosa*	0.64 ± 0.12	2.00 ± 0.35	1.00 ± 0.16
*E. coli*	0.32 ± 0.08	4.00 ± 0.09	2.00 ± 0.33
*C. albicans*	0.18 ± 0.01	4.00 ± 0.16	1.00 ± 0.16

**FIGURE 7:**
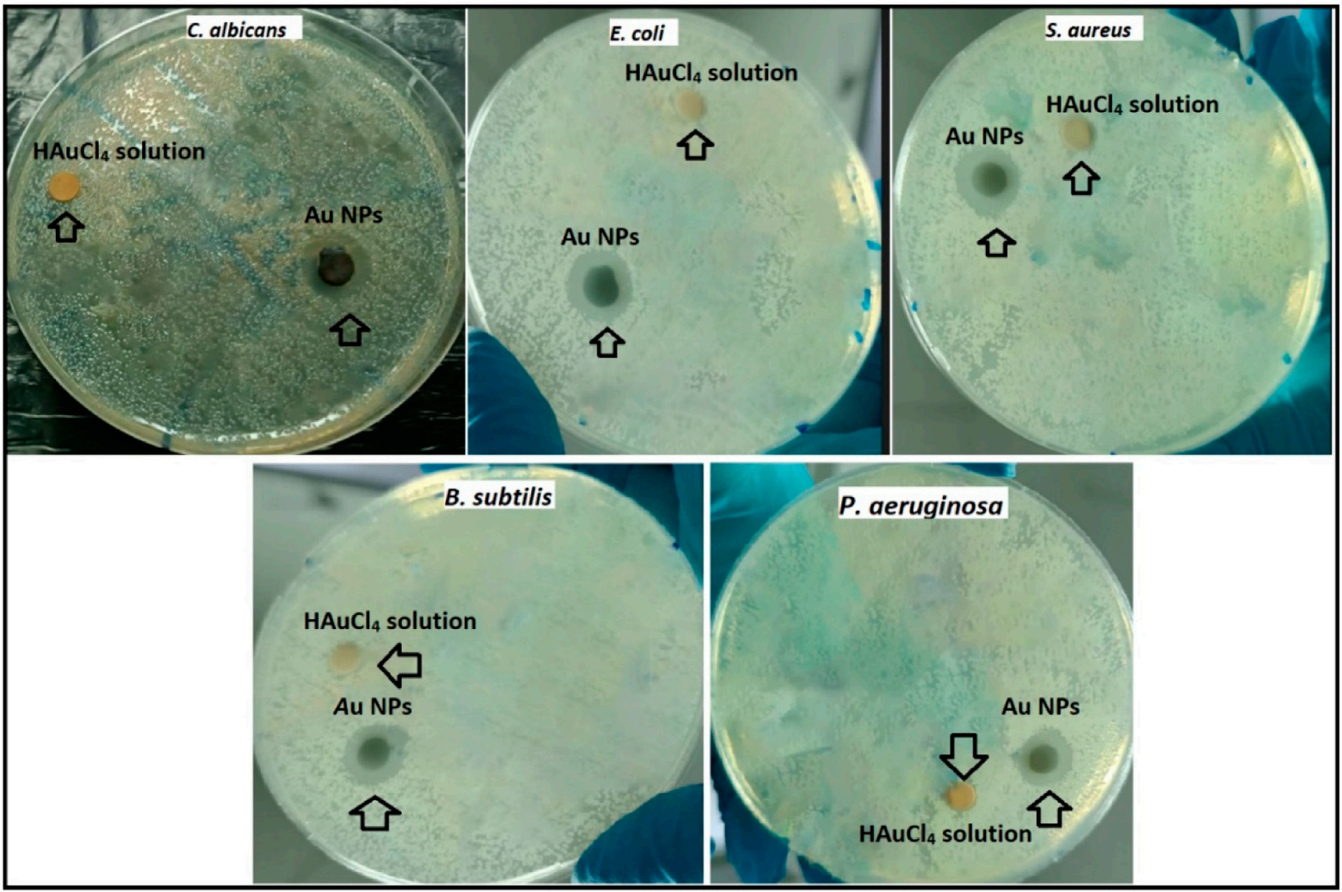
Inhibition zones formed due to suppression of synthesized Au NPs and HAuCl_4_ solution on gram positive *S. aureus* and *Bacillus subtilis*, gram negative *E. coli* and *P. aeruginosa*, and fungus *C. albicans*.

The inhibition zones in the suppression of growth due to the antimicrobial effects of Au NPs synthesized using the disk diffusion method on gram (−) *E. coli* and gram (+) *S. aureus* bacteria as well as fungus *C. albicans* pathogens are given in Figure 7. The inhibition zones formed on *E. coli, P. aeruginosa, S. aureus*, *B. subtilis* and *C. albicans* were measured as 14.14 ± 0.96, 13.24 ± 0.48, 15.84 ± 1.55, 16.83 ± 0.57, and 16.48 ± 0.55 mm, respectively. In the measurement made with HAuCl_4_ solution for comparison and control purposes, a very little suppression was observed with 1.03 ± 0.12, 0.94 ± 0.19, 1.02 ± 0.12, and 1.69 ± 0.52 mm inhibition zones on *S. aureus*, *C. albicans*, *P. aeruginosa,* and *B. subtilis* respectively. However, this suppression was quite low compared to the effect of the synthesized Au NPs. In the findings showing parallelism with the data obtained by microdilution, it was determined that Au NPs synthesized on *B. subtilis* and *C. albicans* were more effective. It was determined that Au NPs synthesized from agricultural waste parts of broccoli, characterized by negative surface charge, stable and spherical morphology with diameters below 100 nm, exhibited antimicrobial effects *in vitro*. Investigation of these effects in vivo applications will be an important step for them to be used as antimicrobial agents for medical applications.

**Scheme 2:**
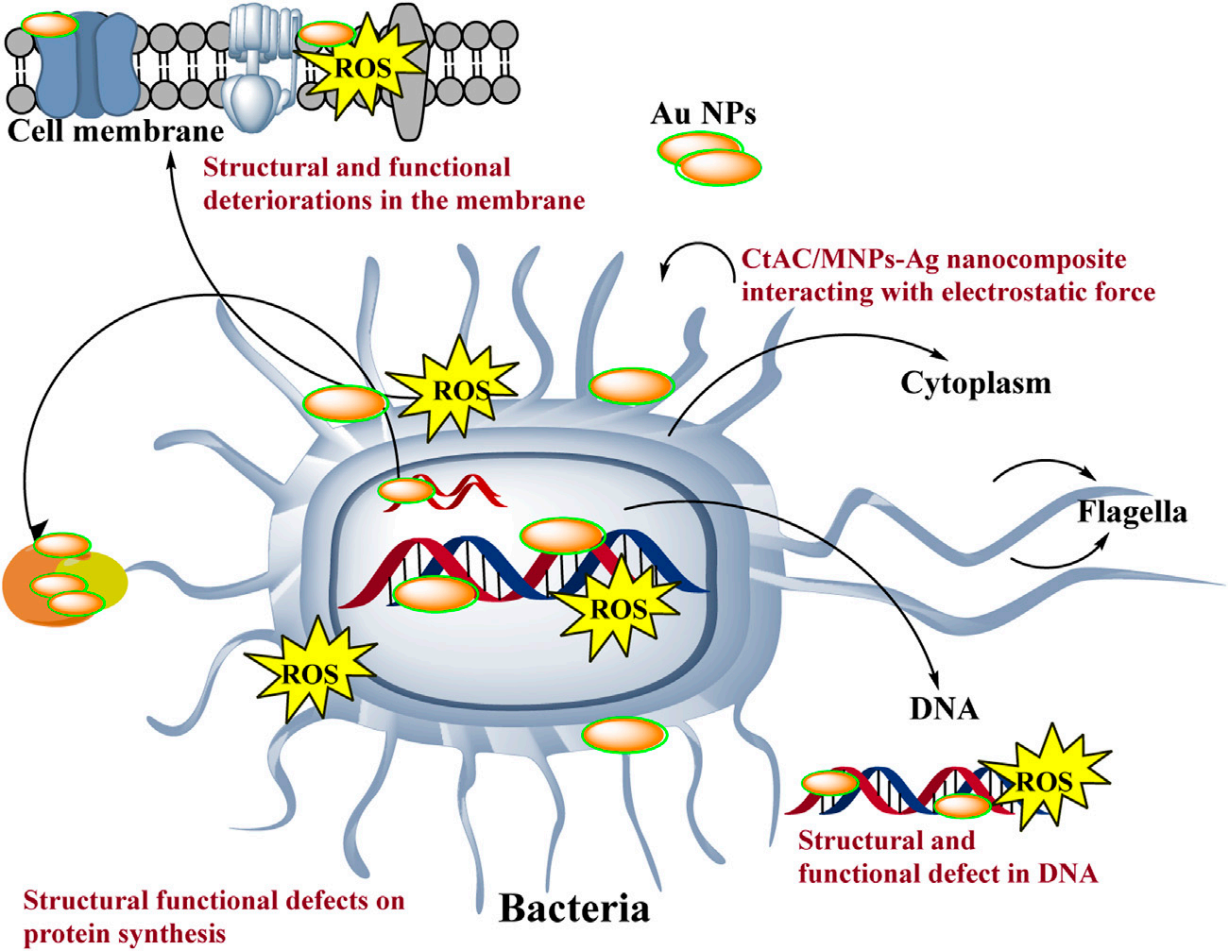
Representative scheme of the antimicrobial effects mechanism of Au NPs by extract components obtained from agricultural parts of Broccoli.

The antibacterial action of Au NPs is influenced by factors such as surface charge, size, and concentration (Jha et al., 2017). Microorganisms have a negative charge, enabling them to interact with positively charged nanoparticles that become ionised in water as a result of the electrostatic force of attraction (Ferreyra Maillard et al., 2018; Babu et al., 2020). Following the interaction with Au NPs, the membrane potential of bacteria undergoes alteration. Exhibits. In addition, Au NPs have detrimental effects on energy metabolism by suppressing ATPase function. Consequently, there is a reduction in the ATP level (Cui et al., 2012; Baran, 2023). In addition, Au nanoparticles also amplify the expression of genes linked to redox processes. As a result, the increase in ROS (reactive oxygen species) causes functional and structural problems in important systems such as energy metabolism. Due to these circumstances that expedite the collapse of biological systems, microorganisms dies (Scheme 2) (Cui et al., 2012; Ahmed et al., 2016; Jha et al., 2017).

### 3.6 Anticancer effects of Au NPs

**FIGURE 8:**
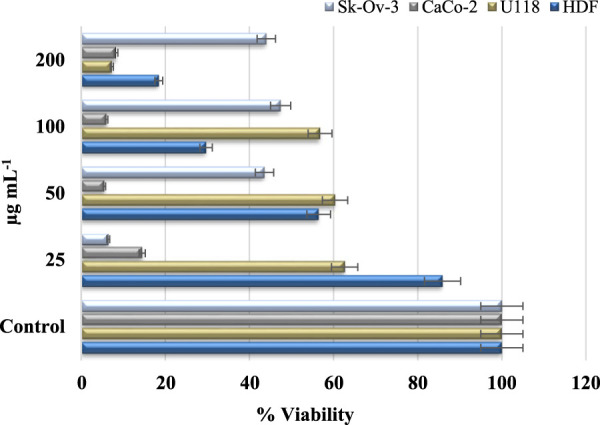
% viability suppressive effect concentrations of Au NPs on healthy cell HDF and cancer cells U118, CaCo-2, and Skov-3.

**Scheme 3:**
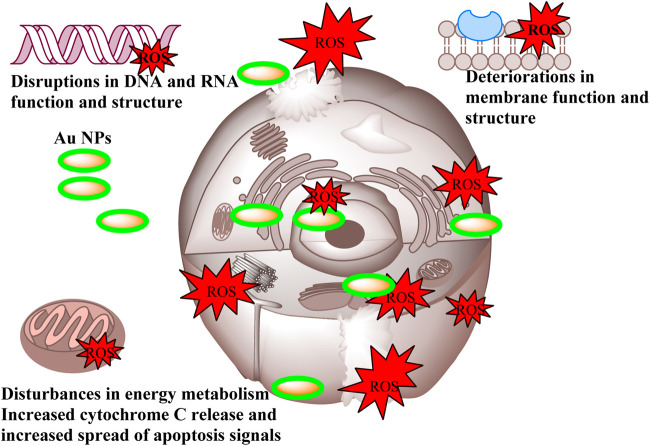
Representative scheme of the anticancer effects mechanism of Au NPs by extract components obtained from agricultural parts of Broccoli.

Cancer is a grave and persistent illness that continues to claim the lives of many individuals in the present day. Extensive research is being conducted to tackle this condition. Research on the quest for anticancer medicines against this serious illness is a highly popular subject (Khatik, 2022). The MTT method was used to investigate the cytotoxic effects of Au NPs synthesised using an extract derived from agricultural waste sections of broccoli. The study focused on the impact of these nanoparticles on the viability of healthy cells (HDF) and cancer cells (CaCo-2, U118, and Skov-3), specifically in terms of lowering their viability percentage. Based on the data shown in Table 5 and Figure 8, it was found that Au NPs had the highest efficacy against Skov-3 and CaCo-2 cancer cells, respectively. The suppression rate achieved was 93.57% and 85.49% at a dose of 25 μg mL^−1^. Furthermore, the identical concentration resulted in a 37.37% reduction in activity on U118. The U118 cancer cells exhibited the highest level of suppression at a concentration of 200 μg mL^−1^, with a suppression rate of 92.72%. However, this dosage also resulted in a suppression of healthy cells by 81.54%. The concentration of 50 μg mL^−1^ exhibited the most optimal impact on CaCo-2. This particular concentration exhibited the highest level of efficacy against these cancer cells. Furthermore, its inhibitory impact on healthy cells was quite minimal. The harmful effects of nanoparticles are significantly influenced by properties like as concentration, size, shape, surface charge, and interaction duration. These characteristics dictate the magnitude, speed, and power of this effect (Remya et al., 2015; Swamy et al., 2015). The diminutive dimensions of Au NPs are a crucial characteristic for medicinal applications. The Au NPs, with an average size of 78.75 nm as shown in Figure 2D, have a significant characteristic when synthesised using the extract derived from the discarded sections of broccoli in agriculture. Furthermore, Figure 3B demonstrates that it possesses just a negative surface charge of −19.7 mV, which plays an essential role in maintaining its stable structure and facilitating its interaction with living systems. Areas of inflammation and tumour growth are characterised by the presence of extensive circulatory arteries that supply nutrients and oxygen. Additionally, these structures have sizable holes. These holes facilitate the accumulation and passage of nanoparticles into these locations (Chen et al., 2021; Oliveira et al., 2024). In addition, Au NPs have the ability to efficiently traverse the cell membrane due to their tiny dimensions. Following migration, Au NPs induce many alterations, including heightened levels of reactive oxygen species (ROS). In addition, Au NPs induce apoptosis through the activation of caspase enzymes. Au NPs have a detrimental impact on mitochondrial permeability. This phenomenon induces atypical states, such as heightened cytochrome c discharge. Subsequently, signals that induce apoptosis are released, leading to the occurrence of cell death as a consequence of these detrimental effects (Scheme 3) (Barabadi et al., 2020; Donga et al., 2020; Webster et al., 2020; Hosny et al., 2022).

**TABLE 5:** % viability rates affecting the viability of Au NPs as a result of their toxic effects on cell lines (n = 3, X̅ ± Sx̅).

Cell Lines	200 µg mL^−1^	100 µg mL^−1^	50 µg mL^−1^	25 µg mL^−1^
HDF	18.46 ± 0.01	29.67 ± 0.01	56.45 ± 0.01	85.90 ± 0.05
Sk-ov-3	44.02 ± 0.01	47.39 ± 0.00	43.59 ± 0.00	6.43 ± 0.01
CaCo-2	8.29 ± 0.01	6.04 ± 0.03	5.51 ± 0.03	14.51 ± 0.01
U118	7.28 ± 0.01	56.76 ± 0.05	60.34 ± 0.01	62.63 ± 0.02

It was observed that Au NPs synthesized via extract had anticancer effects under *in vitro* conditions by suppressing proliferation on cells used in applications. Investigation of these effects under *in vivo* conditions will be an important step for the use of synthesized Au NPs as anticancer agents in medical applications.

A study was conducted to synthesise Au NPs using *Amygdalus communis* leaf extract. The study aimed to investigate the impact of a concentration of 25 µg mL-1 on U118, CaCo-2, Skov-3, and HDF. The study’s findings reported that the viability rates of Au NPs on these cells were 62.6%, 14.5%, 44.0%, and 22.6%, respectively (Baran, 2023).

## 4 Conclusion

Increasing interest in metal nanoparticles synthesised using environmentally friendly methods is due to their extensive range of applications. Metal nanoparticles can be synthesised inexpensively and effortlessly using extracts derived from different plant components through environmentally friendly techniques. In this study, Au NPs were efficiently synthesised using an extract derived from the agricultural waste portions of the Broccoli plant. The synthesis process was rapid, cost-effective, and environmentally sustainable. The novel method used for the extraction preparation procedure yielded favourable outcomes, effectively mitigating time wastage while simultaneously enhancing processing efficiency and reducing energy consumption. The properties of synthesised Au NPs were assessed using various techniques including UV-vis, TEM, FE-SEM, DLS, XRD, AFM, FTIR, TGA-DTA, and EDX. The characterization findings revealed that the Au NPs exhibited a peak absorbance at a wavelength of 553.10 nm, a surface charge of −19.7 mV, an average hydrodynamic size of 78.75 nm, a uniform spherical shape, and demonstrated stability. The inhibitory effects of Au NPs having these characteristics on disease-causing strains and cancer cells were evaluated using microdilution, disk diffusion and MTT techniques. Au NPs were assessed to possess antibacterial, antifungal, and anticancer properties at low concentrations.

Considering the wide range of Au NPs in medical applications, it is thought that the *In vitro* findings of Au NPs synthesized through agricultural wastes of broccoli in an environmentally friendly, biocompatible and stable manner will contribute to the search for pharmacological antimicrobial and anticancer agents by examining them *In vivo*.

## Data Availability

The original contributions presented in the study are included in the article/supplementary material, further inquiries can be directed to the corresponding author.
